# Molecular and Insecticidal Characterization of a Novel Cry-Related Protein from *Bacillus Thuringiensis* Toxic against *Myzus persicae*

**DOI:** 10.3390/toxins6113144

**Published:** 2014-11-06

**Authors:** Leopoldo Palma, Delia Muñoz, Colin Berry, Jesús Murillo, Iñigo Ruiz de Escudero, Primitivo Caballero

**Affiliations:** 1 Instituto de Agrobiotecnología, CSIC-UPNA-Gobierno de Navarra, Campus Arrosadía, Mutilva 31192, Navarra, Spain; E-Mails: leopoldo.palma@unavarra.es (L.P.); iruiz@unavarra.es (I.R.E.); 2 Grupo de Protección Cultivos, Departamento de Producción Agraria, Escuela Técnica Superior de Ingenieros Agrónomos, Universidad Pública de Navarra, Pamplona 31006, Navarra, Spain; E-Mails: dmunoz@unavarra.es (D.M.); jesus.murillo@unavarra.es (J.M.); 3 Cardiff School of Biosciences, Cardiff University, Park Place, Cardiff CF10 3AT, UK; E-Mail: Berry@cardiff.ac.uk

**Keywords:** *Bacillus thuringiensis*, δ-endotoxins, Cry-related protein, parasporins, green peach aphid, *Myzus persicae*

## Abstract

This study describes the insecticidal activity of a novel *Bacillus thuringiensis* Cry-related protein with a deduced 799 amino acid sequence (~89 kDa) and ~19% pairwise identity to the 95-kDa-aphidicidal protein (sequence number 204) from patent US 8318900 and ~40% pairwise identity to the cancer cell killing Cry proteins (parasporins Cry41Ab1 and Cry41Aa1), respectively. This novel Cry-related protein contained the five conserved amino acid blocks and the three conserved domains commonly found in 3-domain Cry proteins. The protein exhibited toxic activity against the green peach aphid, *Myzus persica*e (Sulzer) (Homoptera: Aphididae) with the lowest mean lethal concentration (LC_50_ = 32.7 μg/mL) reported to date for a given Cry protein and this insect species, whereas it had no lethal toxicity against the Lepidoptera of the family Noctuidae *Helicoverpa armigera* (Hübner), *Mamestra brassicae* (L.), *Spodoptera exigua* (Hübner), *S. frugiperda* (J.E. Smith) and *S. littoralis* (Boisduval), at concentrations as high as ~3.5 μg/cm^2^. This novel Cry-related protein may become a promising environmentally friendly tool for the biological control of *M. persicae* and possibly also for other sap sucking insect pests.

## 1. Introduction

*Bacillus thuringiensis* (Bt) is a Gram positive, spore-forming bacterium that synthesizes parasporal crystalline inclusions containing one or more proteins (Cry and Cyt proteins) [1], some of which are toxic to a high number of insect species of the orders Lepidoptera, Diptera and Coleoptera, in addition to a few Hemiptera [2,3,4] and Nematoda [5]. The extremely high insecticidal activity of some Cry proteins is mainly due to their oral toxicity, which is produced after the proteolytic activation of protoxins and their specific binding to receptors located on the plasma membrane of the midgut epithelial cells of susceptible insects [2]. This property makes Bt an environmentally safe and ecologically sound microbial agent for controlling insect pests that ingest the toxins present on the plant surfaces. However, a few Cry proteins have been found to be poorly active against sap-sucking insect pest species such as aphids, whiteflies and plant bugs (Hemiptera). Bt control of these pests, which cause major agricultural losses worldwide [6] and have an extremely rapid ability to develop resistance to classical chemical insecticides [6,7,8], may be useful for developing integrated pest management strategies. A handful of Cry proteins have been found to be weakly to moderately active against hemipterans in artificial diet feeding assays [4,9,10]. Only recently, a Bt crystal protein and a binary vegetative insecticidal protein (Vip1/Vip2) with aphidicidal potential have been reported [9,11]. Due to their feeding behaviour on the phloem sap of plants, hemipterans can only ingest significant amounts of Cry proteins when they are transported in the phloem, e.g., if they are efficiently expressed in transgenic Bt plants. So far, only two Bt Cry proteins have been expressed in Bt-plants (cotton and wheat), which resulted in deleterious effects for the development and survival of two hemipteran species: the cotton pest *Lygus hesperus* (Knight) (Hemiptera: Miridae) [12] and the wheat aphid *Schizaphis graminum* (Rondani) (Hemiptera: Aphididae) [13]. Bt populations naturally present in the soil and the phylloplane often synthesize Cry proteins with unknown biological activities, which are estimated to account for more than 90% of the strains of this bacterium in natural environments [14,15]. These are generally referred to as non-insecticidal isolates due to their lack of toxicity against lepidopterans, dipterans and coleopterans [14,16]. However, some parasporal inclusions produced by such strains and with no known insecticidal activity exhibit selective toxic activity against human cancer cells and have been designated as parasporins [14,15,17]. Subsequently, parasporins have been given alternative Cry designations, which will be used throughout this report. The existence of several Cry proteins with biological roles other than insecticidal, e.g. the toxicity of several parasporins against human cancer cells [14] and a human pathogenic protozoan [18], led us to hypothesize that Cry proteins active against hemipterans may exist in greater numbers than previously recognized. In this study, we report the identification, cloning and molecular characterization of a novel Cry-related protein, and its toxic activity against the green peach aphid, *Myzus persicae* (Sulzer) (Hemiptera: Aphididae).

## 2. Results

### 2.1. Draft Genome Sequence of Strain H1.5

A total of 782 contigs were obtained, totalling 5,958,193 bp with 35.2% G+C content and 6130 predicted CDSs (coding sequences), including the full-length coding sequence of the novel *cry*-related gene among other CDSs exhibiting homology to different insecticidal proteins, namely 7 CDSs were homologous to Cry proteins, two CDSs homologous to Cyt proteins, two CDSs homologous to Vip1 proteins and two CDSs homologous to Vip2 proteins.

### 2.2. Molecular Characterization of the Novel Cry Gene

**Figure 1:**
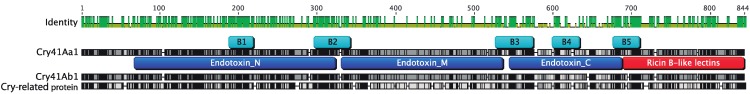
Alignment of parasporins (Cry41Aa1, Cry41Ab1) and the Cry-related protein deduced amino acid sequence. Light-blue bars (B1 to B5) indicate the conserved five amino acid blocks, Endotoxin (N, M and C) the three-conserved domains and ricin B-like lectin indicates the *C*-terminal conserved lectin domain found in the three protein sequences.

The predicted *cry*-related gene (accession no. KJ427833) was present in a contig that only contained this coding sequence, which is 2397 bp long and encoding a protein of 799 amino acids and predicted molecular weight of 89 kDa. Its predicted protein sequence exhibited ~19% pairwise identity to the 95-kDa-aphidicidal protein (sequence 204) described in patent US 8318900 (accession no. AGA40064) [9] and ~40% pairwise identity to cancer cell killing Cry proteins (parasporins Cry41Ab1 and Cry41Aa) [19] (Figure 1). It contained the five conserved blocks found in 3-domain Cry proteins [1] and in parasporins Cry41Aa1 and Cry41Ab1 [19] exhibiting 84.9%, 74.1%, 60.4%, 81.5% and 90% pairwise identity from blocks 1–5, respectively. The Cry-related protein also had the three Cry typical conserved domains endotoxin_N (InterPro accession no. IPR005639), endotoxin_M (InterPro accession no. IPR015790) and delta_endotoxin_C (InterPro accession no. IPR005638) present in other known Cry proteins. It also possessed a *C*-terminal ricin B-like lectin conserved domain between amino acids 652 and 799, which is also present in parasporins Cry41Aa1 and Cry41Ab1 (Figure 1), but absent in the 95-kDa-aphidicidal protein from patent US 8318900 (data not shown).

**Figure 2:**
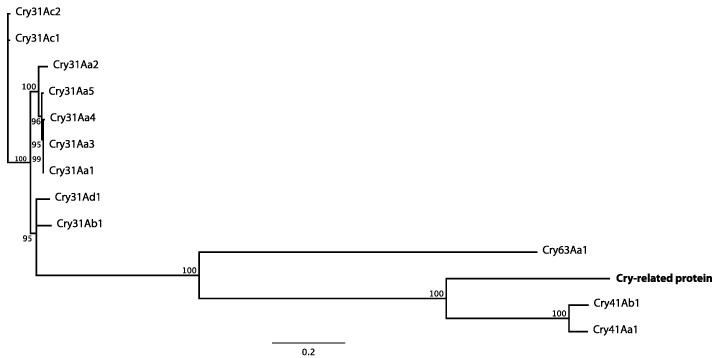
Dendrogram showing the relationship of a novel Cry-related protein to other parasporin (Cry) proteins. Bootstrap values are indicated at the nodes (100 replicates).

A phylogenetic analysis comparing the deduced Cry-related amino acid sequence against known parasporin proteins was performed. The dendrogram showed that the Cry-related protein remained grouped with parasporins Ps6Aa1 (Cry63Aa1) [20], Ps3Aa1 (Cry41Aa1) and Ps3Ab1 (Cry41Ab1) separately from the rest of parasporins (Figure 2).

### 2.3. Protein Expression of the Cry-Related Gene

**Figure 3:**
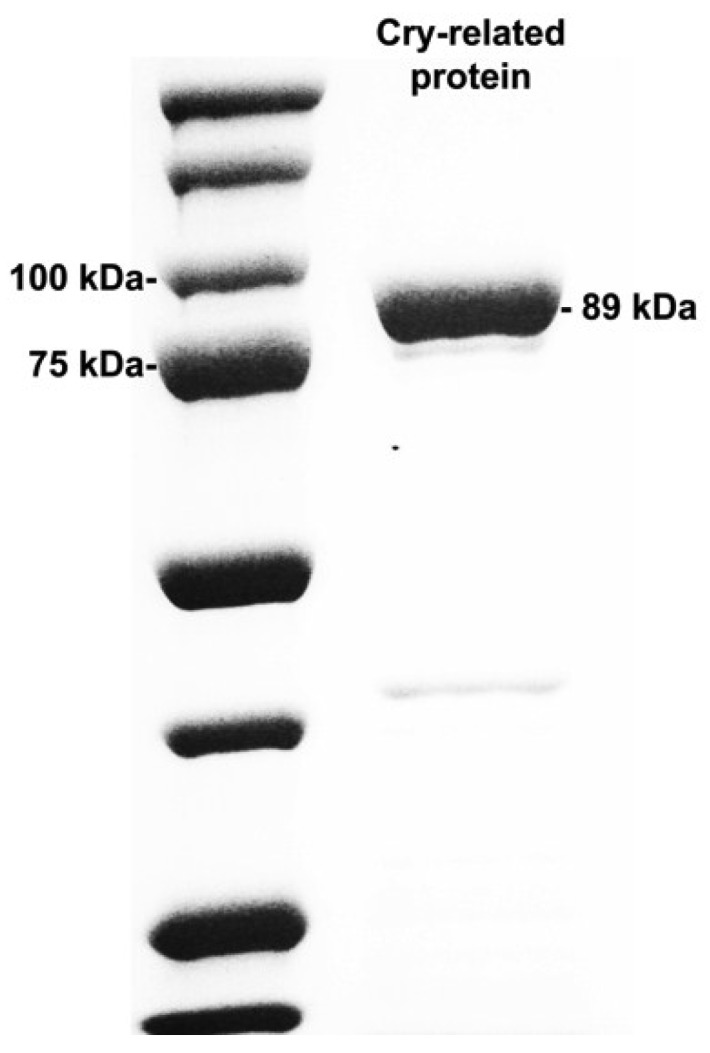
Sodium dodecyl sulfate-polyacrylamide gel electrophoresis (SDS-PAGE) analysis of the Cry-related recombinant protein expressed in *E. coli* (right lane). A molecular weight marker (Precision Plus Protein Standards, Bio-Rad) was electrophoresed along with the sample and the sizes of the related fragments are indicated to the left of the panel.

SDS-PAGE analysis showed that the novel Cry-related protein was successfully expressed in *E. coli* (Figure 3) and produced a major protein of the expected size (approx. 89 kDa) that was found in both the soluble fraction (active protein) and the pellet removed by centrifugation (data not shown), indicating that the recombinant Cry-related protein was produced in both soluble and insoluble forms (inclusion bodies) as result of overexpression.

### 2.4. Insecticidal Activity of the Novel Cry-Related Protein

The novel Cry-related protein showed aphidicidal activity against the green peach aphid, *Myzus persicae*. In the preliminary bioassays, mortality was higher than 75% with the two concentrations used (100 and 1000 µg/mL) and, therefore, the LC_50_ value was estimated using four concentrations, 20, 25, 32 and 40 µg/mL, that killed 16% ± 6%, 23% ± 5%, 55% ± 7% and 83% ± 4% of the aphids, respectively. The concentration-mortality responses of the Cry-related protein for *M. persicae* fitted a regression line. The interaction between host population and log [protein dose] was not significant with the protein, exhibiting a median lethal concentration (LC_50_) of 32.7 μg/mL (Table 1). In contrast, a single concentration of ~3.5 μg/cm^2^ resulted in no larvicidal activity or impaired growth against any of the five lepidopteran species screened (*H. armigera*, *M. brassicae*, *S. exigua*, *S. frugiperda* or *S. littoralis*).

**Table 1:** Toxicity of the Cry-related *B. thuringiensis* protein against second-instar *M. persicae* nymphs.

Treatment	LC_50_ (µg/mL)	Regression line		Goodness of fit value	
		Slope ± SE	a * ± SE	χ^2^	d.f.
Cry-related protein	32.7	10.3 ± 1.4	−10.6 ± 2.1	0.55	2

* a: intercept of the regression line.

## 3. Discussion

In the present study, we report the molecular and insecticidal characterization of a novel Cry-related protein encoded into the genome sequence of Bt strain H1.5. This genome sequence exhibited typical Bt genome features found in other sequenced Bt strains (*i.e*., genome size, % G+C, number of predicted CDSs, *etc*.); however, this strain also harbours several different and potentially novel insecticidal genes in which their pairwise identity (data not shown) strongly suggest that they are susceptible to be protected by patent applications. The novel Cry-related protein contained the five amino acid conserved blocks and the three conserved domains (Endotoxin_N, Endotoxin_M and delta_endotoxin_C) commonly present in other three-domain Cry toxins (Figure 1). Interestingly, it also possessed a *C*-terminal ricin B-like lectin conserved domain between amino acids 652 and 799 (Figure 1), which was absent in the aphidicidal protein of 95 kDa (US 8318900) [9]. The novel aphidicidal Cry-related protein and its phylogenetic relationship with parasporins Cry41Aa1 and Cry41Ab1 may suggest its possible activity against cancer cells, a possibility that is currently under investigation [19]. Sap-sucking insect pests (aphids, whiteflies and plant bugs) have shown very low susceptibility to some Cry proteins [6,21,22] with the lowest reported LC_50_ values ranging from 70 to 100 μg/mL for Cry4Aa and higher than 500 μg/mL for Cry3A and Cry11Aa against the pea aphid *Acyrtoshiphon pisum* (Harris) (Hemiptera: Aphididae) [4]. Multiple factors may be responsible for the low toxicity of Cry proteins against hemipteran pests relative to lepidopteran pests, the most important being the gut pH, which is acidic in aphids and alkaline in lepidopterans, and the abundance and type of proteolytic enzymes, cysteine proteases in aphids and mainly serine proteases (trypsin and chymotrypsin) in lepidopterans. This explains the higher toxicity of trypsin-pre-activated Cry3A, Cry4A and Cry11A against *A. pisum* [4]. The novel Cry-related protein exhibited two-fold higher toxicity against the green peach aphid, *M. persicae*, with an LC_50_ value of 32.7 µg/mL, than Cry4Aa for *A. pisum*, in the range 70–100 μg/mL [4]. However, and compared to the 95-kDa aphidicidal protein, which killed 80% *L. hesperus* at approximately 50 μg/mL showing also some (not quantified) toxic activity against *Aphis glycines* (Matsumura) (Hemiptera: Aphididae) [9], the novel Cry-related protein exhibited a similar performance, killing 83% of *M. persicae* aphids at 40 μg/mL. The steep regression line slope of Cry-related protein for *M. persicae* was noticeable, and was much higher than the regression line slopes of other Bt proteins, which are usually between 0 and 4 [23,24,25], indicating that small changes in protein concentrations have a dramatic effect on aphid mortality. The ricin B-like lectin domain might be involved in the toxicity of this protein against *M. persicae*. Lectins are carbohydrate-binding proteins that play a role in plant defences against herbivorous insects and affect several insect physiological processes by binding to glycoproteins of the gut membrane being particularly antinutritional and toxic for hemipterans [6,26]. For example, Cry1Ac protein, upon fusion to the nontoxic ricin B-chain galactose/*N*-Acetylgalactosamine binding lectin, increased toxicity against susceptible and resistant insects but also against the insect pest *Cicadulina mbila* (Naude) (Hemiptera: Cicadellidae) [27], which is not susceptible to natural Cry1Ac proteins [4]. The purified (soluble) Cry-related protein was not toxic to any of the five lepidopteran species tested (*H. armigera*, *M. brassicae*, *S. exigua*, *S. frugiperda* and *S. littoralis*) while the specificity bases for the activity against *M. persicae* remain unknown. Crystal proteins are ingested as non-toxic protoxins, solubilized and proteolytically transformed to active protease-stable fragments into the midgut of susceptible insects [1,28]. Defects in activation or absence of specific midgut proteases can lead to the lack of activity for a given Bt toxin. For instance, in the lepidopterans *Plodia interpunctella* (Hübner) and *Heliothis virescens* (Fabricius), resistance to Cry1A was produced because midgut proteases failed in proteolytically activating the protoxin [29]. Because of the *per os* mode of action of Bt and the sap-sucking feeding behaviour of aphids, development of genetically modified crops expressing aphidicidal Cry proteins is a potential method for Bt-control of aphids. In addition, toxins should be present in the plant phloem in lethal quantities. Previous studies have demonstrated the effective action of different aphidicidal proteins expressed in transgenic plants under the control of powerful promoters such as the CaMV35S constitutive plant viral promoter (cauliflower mosaic virus 35S promoter) or the phloem-specific sucrose synthase promoter *Asus1* from *Arabidopsis thaliana* [26,30]. For example, Yu and Wei (2008) successfully constructed a transgenic wheat plant that stably expressed the Cry1Ac protein and the PTA agglutinin under the control of the CaMV35S promoter, which confers resistance to the wheat aphid *S. graminum* and the oriental armyworm *Mythimna separata* (Walker) (Lepidoptera: Noctuidae), producing significant reductions in the survival rate, development, fecundity and biomass in both species [13]. Moreover, Baum *et al.* (2012) constructed a transgenic cotton plant expressing the Cry51Aa2 protein, also controlled by the CaMV35S promoter, that negatively affected the survival and development of the plant bug *L. hesperus* [12]. A transgenic Bt-oilseed rape expressing a truncated synthetic version of the *cry1Ac* gene (driven also by the CaMV35S promoter) has been used to analyse the bioavailability of the protein against the aphid, *M. persicae*, which is a non-target species for the Cry1Ac protein [21]. The protein was detected in the plant phloem at very low concentrations (2.7 parts-per-billion), suggesting the potential value of using highly aphidicidal Cry proteins to target these insects. As an alternative to transgenic applications, it has been shown that some Bt strains can be taken up endophytically by plants [31] and that application of Bt strains to cut leaf stems could result in toxicity to *Aphis gossypii* (Glover) (Hemiptera: Aphididae) [32]. Development of this technology may contribute with the use of aphidicidal Bt strains into integrated pest management strategies. Here, the novel aphidicidal Cry-related protein has successfully proven its toxicity against the green peach aphid *M. persicae*, and may become a promising environmentally-friendly tool for the biological control of this hemipteran pest.

## 4. Materials and Methods

### 4.1. Bacterial Strains and Plasmids

The Bt H1.5 strain, from the Bt collection held at the Universidad Pública de Navarra (Pamplona, Spain) [33,34] and the *Escherichia coli* DH5α strain were cultured in Luria-Bertani (LB) medium (1% tryptone, 0.5% yeast extract, and 1% NaCl, pH 7.0) at 28 °C and 37 °C, respectively. *E. coli* DH5α was used as a host strain for transformation whereas *E. coli* BL21(DE3) was used for protein expression and grown in 2× YT medium (1.6% tryptone, 1% yeast extract, and 0.5% NaCl, pH 7.0) at 37 °C. The vector pGEM-T Easy (Promega, Madison, WI, USA) was used for routine cloning of PCR products and the expression vector pET-28b(+) (Merck Millipore, Darmstadt, Germany) was used for the production of recombinant proteins.

### 4.2. Genome Sequencing

Total DNA (chromosome and plasmids) from Bt strain H1.5 was isolated using the Wizard Genomic DNA Purification Kit (Promega, Madison, WI, USA) following the manufacturer’s instructions for DNA isolation from Gram-positive bacteria. The purified DNA was then used to construct a pooled Illumina library and sequenced using a HiSeq 2000 Sequencing System (Illumina Sequencing, San Diego, CA, USA) in a single read mode with a read length of 50 bases (GATC Biotech, Constance, Germany).

### 4.3. Computational Analysis of DNA and Protein Sequences

The millions of reads produced by sequencing were assembled using CLC Genomic Workbench 6.0 (Qiagen, Aarhus, Denmark) with the *de novo* assembly tool and default parameters. The resultant contigs were then analysed with BLAST [35] using a custom insecticidal toxin database constructed with insecticidal protein sequences obtained from public databases and the BtToxin_scanner [36]. Multiple sequence alignments, phylogenetic trees, conserved domain searches, cloning and primer design, were performed using suitable tools included in Geneious Pro v6.1.4 (Biomatters Ltd., Auckland, New Zealand) [37].

The multiple sequence alignment was performed including amino acid sequences of Cry-related protein and parasporin (Cry) proteins using Geneious Alignment tool and default parameters. A Neighbor-Joining phylogenetic inference was performed on the multiple amino acid alignment with the Geneious Tree Builder tool by using Jukes-Cantor as genetic distance model. The support for nodes in the tree was obtained from 100 bootstrap iterations.

### 4.4. Amplification and Cloning of the Novel Cry-Related Gene Sequence

For the amplification of the full-length *cry*-related gene sequence, a pair of primers was designed based on the sequences upstream of the start (ATG) and downstream of the stop (TAA) codons of the gene. The PCR reaction was performed in a 25 μL (final volume) mixture containing 5 μL 5× reaction buffer, 10 mM dNTPs, 6.3 pmol forward and reverse primers, 0.5 U proof reading PrimeSTAR HS DNA polymerase (Takara Bio, Otsu, Japan) and 100 ng total DNA. PCR was performed using the following cycling conditions: 4 min initial denaturation at 94 °C, 35 amplification cycles (1 min denaturation at 94 °C, 1 min annealing at 52 °C and 2.5 min extension at 72 °C) with a final extension step at 72 °C for 10 min. The blunt-ended, ~2.4 kb PCR product was agarose purified using NucleoSpin Extract II kit (Macherey-Nagel, Düren, Germany), end modified by an A-tailing procedure and ligated into pGEM-T Easy according to the manufacturer’s instructions (Promega, Madison, WI, USA). The ligated insert was then cloned into *E. coli* DH5α using standard procedures [38]. Plasmid DNA was purified using the NucleoSpin Plasmid kit (Macherey-Nagel, Düren, Germany) and the corresponding DNA sequences obtained with an automatic Applied Biosystems 3730×I nucleotide sequence analyzer (Sistemas Genómicos, Valencia, Spain). The sequence obtained was analysed from at least two different H1.5 colonies to ensure that no mismatches were present in the wild-type sequence. This allowed the design of another full-length pair of primers from the start to the stop codon including an upstream 5'*Bam*HI (5'-GGATCCGATGAACCAAAATTATAACAAC-3') and a downstream 3' *Sal*I (5'-GTCGACTTATAACTTATTCAGTTTG-3') restriction sites. The insert was then ligated into pGEM-T easy and transformed into *E. coli* DH5α. Plasmid was purified using the NucleoSpin Plasmid kit (Macherey-Nagel, Düren, Germany), digested with B*am*HI and *Sal*I restriction enzymes, purified from the agarose gel, ligated into predigested pET-28b(+), and cloned into *E. coli* BL21(DE3). A clone of the recombinant strain was selected and sequenced to verify the correct *N*-terminal fusion to the polyhistidine-tag encoded in the pET-28b(+) expression vector.

### 4.5. Protein Expression and Purification

The recombinant *E. coli* BL21(DE3) strain harbouring the novel *cry*-related gene was pre-cultured overnight at 37 °C with vigorous shaking at 200 rpm (MaxQ 4000 orbital shaker, ThermoFisher, Waltham, MA, USA) in 2× YT medium containing 50 μg/mL kanamycin. This pre-culture was diluted 1/25 in 500 mL 2× YT medium containing 50 μg/mL kanamycin and further incubated at 37 °C with vigorous agitation (250 rpm) up to an OD_600_ of between 0.7 and 1.0. Expression was induced immediately by adding isopropyl-β-D-1-thiogalactopyranoside (IPTG, Bioline, London, UK) to a final concentration of 1 mM and incubation for 1–3 h. Samples were centrifuged at 5000 g for 15 min at 4 °C, and the resulting pellet weighed and resuspended with 3 mL sonication buffer (50 mM NaH_2_PO_4_ pH 8.0, 300 mM NaCl, 3 mg/mL lysozyme, 25 U Benzonase (Novagen, Billerica, MA, USA) and 100 μM phenylmethylsulfonyl fluoride (PMSF, Calbiochem, Darmstadt, Germany) per gram of pellet. Samples were further incubated at 37 °C for 30 min and sonicated on ice water with a Branson analog sonifier 250 (Branson Ultrasonics Corporation, Danbury, CT, USA) by applying two 1 min pulses with constant duty cycle at 60 W, separated by a 1 min cooling period. Insoluble material was pelleted by centrifugation at 12,000 *g* for 30 min at 4 °C and the soluble cellular fraction filtered through sterile 0.45 and 0.22 μm syringe filters. Protein purification was performed at room temperature using Protino Ni-TED 2000 Packed Columns according to the manufacturer’s instructions (Macherey-Nagel, Düren, Germany). Once the bound polyhistidine-tagged protein was eluted, the buffer exchange procedure was performed immediately at room temperature with Milli-Q water and GE Healthcare PD-10 desalting columns. This step also prevents the potential toxic effects of imidazole [39,40] to the insects tested in bioassays. The resultant expressed protein was then concentrated by lyophilization, resuspended in Milli-Q water, and analyzed by sodium dodecyl sulfate-polyacrylamide (Bio-Rad, Alcobendas, Spain) gel electrophoresis (SDS-PAGE) stained with Coomassie brilliant blue R-250 (Sigma-Aldrich, Dorset, UK) [38]. The protein concentration was quantified by the Bradford method [41].

### 4.6. Insect Rearing and Bioassays

A colony of *M. persicae* (Sulzer) was started from a single virginoparous female collected in a pepper field (El Encín, Alcalá de Henares, Spain) in 1989. This aphid species was reared on pepper plants (*Capsicum annuum* L.) in controlled environmental conditions, at 25 °C, 60% ± 5% RH (relative humidity) and a 16:8 h (light:dark) photoperiod. The five lepidopteran insects tested, *Helicoverpa armigera* (Hübner), *Mamestra brassicae* (L.), *Spodoptera exigua* (Hübner), *Spodoptera frugiperda* (J.E. Smith), and *Spodoptera littoralis* (Boisduval) belong to the Noctuidae family and have been reared for more than 25 generations on artificial diet [42] under controlled conditions (25 °C, 60% ± 5% RH, and 16:8 h light:dark photoperiod) at the insectary facilities of the Universidad Pública de Navarra. For the bioassays with *M. persicae*, cohorts of 300 second-instar nymphs were obtained from gravid females during a two-day period prior to the experiment. To evaluate the insecticidal activity of the novel Cry-related protein, the purified (soluble) recombinant protein was incorporated into a liquid diet containing 20% (*w*/*v*) sucrose in Milli-Q water. Preliminary assays to estimate protein activity were performed twice with two protein concentrations (100 and 1000 μg/mL) each time. The median lethal concentration (LC_50_) was determined using four different protein concentrations (20, 25, 32 and 40 μg/mL) that were estimated to kill between 5% and 95% of the tested insects. The bioassay was performed eight times. Groups of 15 second-instar nymphs were placed inside a cylindrical plastic cage without a lid (3 cm diameter × 1.5 cm height). The cages were covered with a Parafilm (Bemis, Neenah, WI, USA) layer, on which 100 µL drops of protein-containing diet or protein-free diet (as negative controls) were loaded and confined using a second Parafilm layer. Water was used instead of protein dilutions in negative controls. Aphids were allowed to feed on the liquid diet through the Parafilm membrane. Bioassays were conducted at 25 °C, 60% ± 5% RH, and a 16:8 h (light:dark) photoperiod. Mortality was recorded after 72 h. Concentration-mortality results were subjected to Probit analysis [43] and expressed as the median lethal concentration (LC_50_) in μg/mL. For the bioassays with lepidopterans, groups of 24 neonate larvae of *H. armigera*, *M. brassicae*, *S. exigua*, *S. frugiperda* and *S. littoralis* were used. A single dose of ~3.5 μg/cm^2^ of the purified (soluble) recombinant protein diluted in water was poured onto 9 cm petri dishes (200 μL/plate) containing a layer of solidified artificial diet covering the entire dish base, and allowed to dry in sterile conditions. Water was used instead of protein dilutions in negative controls. Bioassays were conducted at 25 °C, 60 ± 5% RH, and a 16:8 (light/dark (h)) photoperiod and mortality was recorded after seven days.

## 5. Conclusions

In this study, a novel *cry*-related gene was successfully identified from Bt strain H1.5, cloned and expressed in *E. coli*. The insecticidal activity of the purified recombinant protein was tested in bioassays against several lepidopteran pest species and the green-peach aphid *M. persicae*. This novel Cry-related protein showed the lowest LC_50_ value (32.7 μg/mL) reported to date for a Cry protein against an hemipteran pest, which are not in general very susceptible to the toxic activity of the Cry toxins produced by Bt.
